# Over Restriction of Dietary Protein Allowance: The Importance of Ongoing Reassessment of Natural Protein Tolerance in Phenylketonuria

**DOI:** 10.3390/nu11050995

**Published:** 2019-04-30

**Authors:** Alex Pinto, Manuela Ferreira Almeida, Anita MacDonald, Paula Cristina Ramos, Sara Rocha, Arlindo Guimas, Rosa Ribeiro, Esmeralda Martins, Anabela Bandeira, Richard Jackson, Francjan van Spronsen, Anne Payne, Júlio César Rocha

**Affiliations:** 1 Faculty of Health & Human Sciences, University of Plymouth, Plymouth PL6 8BH, UK; alex.pinto@nhs.net (A.P.); anne.payne@plymouth.ac.uk (A.P.); 2 Centro de Genética Médica, Centro Hospitalar Universitário do Porto (CHUP), 4099-028 Porto, Portugal; manuela.almeida@chporto.min-saude.pt (M.F.A.); pcramos.nut@gmail.com (P.C.R.); 3 Centro de Referência na área de Doenças Hereditárias do Metabolismo, Centro Hospitalar Universitário do Porto-CHUP, 4099-001 Porto, Portugal; saraisabelrocha@gmail.com (S.R.); arlguimas@gmail.com (A.G.); rocrff@gmail.com (R.R.); esmeralda.g.martins@gmail.com (E.M.); anabela.ol.bandeira@sapo.pt (A.B.); 4 Unit for Multidisciplinary Research in Biomedicine, Abel Salazar Institute of Biomedical Sciences, University of Porto-UMIB/ICBAS/UP, 4050-313 Porto, Portugal; 5 Birmingham Women’s and Children’s Hospital, Birmingham B4 6NH, UK; anita.macdonald@nhs.net; 6 Cancer Research UK Liverpool Cancer Trials Unit, University of Liverpool, Liverpool L69 3GL, UK; R.J.Jackson@liverpool.ac.uk; 7 Beatrix Children’s Hospital, University Medical Center Groningen, University of Groningen, 9713 Groningen, The Netherlands; f.j.van.spronsen@umcg.nl; 8 Centre for Health Technology and Services Research (CINTESIS), 4200-450 Porto, Portugal

**Keywords:** BH4, natural protein tolerance, phenylketonuria, phenylalanine

## Abstract

Phenylalanine (Phe) tolerance is highly variable in phenylketonuria (PKU) and rarely described in patients aged ≥12 years. Patients ≥12 years of age with PKU were systematically challenged with additional natural protein (NP) if blood Phe levels remained below 480 µmol/L (i.e., upper target blood Phe level for patients aged ≥12 years using Portuguese PKU guidelines). In PKU patients, NP tolerance was calculated at baseline and a median of 6 months after systematic challenge with NP whilst patients were maintaining a blood Phe ≤480 μmol/L. Anthropometry was assessed at both times. Routine blood Phe levels were collected. We studied 40 well-controlled PKU patients (10 hyperphenylalaninemia (HPA), 23 mild and 7 classic PKU), on a low-Phe diet with a mean age of 17 years (12–29 years). Median daily NP intake significantly increased between assessments (35 vs. 40 g/day, *p* = 0.01). Twenty-six patients (65%) were able to increase their median NP intake by a median 12 g/day (2–42 g)/day and still maintain blood Phe within target range. Out of the previous 26 patients, 20 (77%) (8 HPA, 11 mild and 1 classical PKU) increased NP from animal sources (e.g., dairy products, fish and meat) and 6 patients (23%) (3 mild and 3 classical PKU) from plant foods (bread, pasta, potatoes). Median protein equivalent intake from Phe-free/low-Phe protein substitute decreased (0.82 vs. 0.75 g/kg, *p* = 0.01), while median blood Phe levels remained unchanged (279 vs. 288 μmol/L, *p* = 0.06). Almost two-thirds of patients with PKU tolerated additional NP when challenged and still maintained blood Phe within the national target range. This suggests that some patients with PKU treated by a low-Phe diet only may over restrict their NP intake. In order to minimise the burden of treatment and optimise NP intake, it is important to challenge with additional NP at periodic intervals.

## 1. Introduction

Phenylketonuria (PKU, OMIM 261600) is an autosomal recessive disorder defined by a deficiency of phenylalanine hydroxylase (PAH, EC 1.14.16.1) resulting in a complete or partial inability to convert phenylalanine (Phe) into tyrosine [1]. The management of PKU is based on a low-Phe diet supplemented with a low-Phe/Phe-free protein substitute [2]. In Portugal, national PKU guidelines advise to maintain blood Phe levels between 120 to 360 μmol/L in children aged <12 years and 120 to 480 μmol/L in children above 12 years [3]. 

Usually, most children with classical PKU tolerate less than 500 mg of Phe per day [4]. Phe tolerance has been defined as the amount of Phe a patient can tolerate whilst maintaining blood Phe concentrations within a target range [5]. There are several factors that can influence Phe tolerance such as severity of patient’s phenotype, patient’s age, target blood Phe levels, dosage and adherence to protein substitute [6]. 

In PKU, lifetime natural protein (NP) tolerance is neither defined or described. Van Spronsen et al. demonstrated that Phe tolerance may increase with age, with Phe tolerance at age 2, 3 and 5 years being a reliable predictor of tolerance at 10 years of age [7]. Phe tolerance data is scarce in adults, but it is believed that many adult patients tolerate more Phe then prescribed by their health professionals. Van Rijn M. et al. [8] has shown in six well-controlled patients that an incidental additional Phe intake of 100% and even 200% extra for one week each was tolerated without affecting blood Phe control. It is possible that many adult patients with PKU have a higher intake and may still achieve blood Phe within the targets of the guidelines. 

A study performed in a small group (*n* = 8) of not well-controlled adult patients with PKU showed that being male and having lower body mass index (BMI) was more predictive of higher protein tolerance (when expressed as Phe mg/kg/day) than genotype alone [9]. Overweight patients (BMI >25 to 28 kg/m^2^) did not increase Phe tolerance compared with their tolerance at age 5 years when Phe tolerance was expressed relative to actual body weight [9]. 

Maximising NP intake is essential. Over-restriction of NP intake may contribute to poor growth, anorexia or osteopenia [4] and sub-optimal growth has been noted particularly in early childhood and adolescence in PKU [10,11], with adults under-achieving their final height. Head circumference increase has been shown to align with NP intake [12]. Fat-free mass and improved body composition was also associated with higher intakes of NP intake [13,14], particularly when NP intake was >0.5 g/kg/day [13]. A new treatment option, sapropterin (BH4), increases NP tolerance in around 20–50% of responsive patients [15]. An important measure of its effectiveness is that it increases NP intake by at least 100% [6]. This treatment requires the reassessment of NP tolerance before commencing testing for BH4 responsiveness. 

The aim of this retrospective longitudinal study was to assess any changes in NP intake, when challenged systematically, in a group of well-controlled patients with PKU on diet treatment only above 12 years of age. 

## 2. Materials and Methods 

### 2.1. Project Design

The European PKU guidelines [6] recommend that all patients with PKU should be considered for BH4 responsiveness and this was the policy for patients with PKU treated in Centro Hospitalar Universitário do Porto. In the months before BH4 responsive testing, patients were systematically challenged with additional NP if blood Phe levels were <480 µmol/L. 

We performed a retrospective longitudinal study examining NP tolerance at baseline and at a median of 6 months (range 2–10) after systematic challenge with extra NP between assessments, maintaining blood Phe ≤ 480 µmol/L. 

### 2.2. Data Collection

Data was collected for anthropometric measurements (weight, height, BMI), dietary intake based on 24-h recall (NP (g/day)), protein equivalent intake from protein substitute (g/kg) and total protein intake (g/day) at each assessment.

### 2.3. Anthropometry

Measurements were performed by J.C.R. and M.F.A. following the same procedures. Each subject’s height and weight were measured when wearing lightweight clothes, without shoes, using a stadiometer (Seca gmtbh & co., Hamburg, Germany) (measured to the nearest millimetre) and a Seca^®^ mechanic weight scale (Seca gmtbh & co., Hamburg, Germany) (measured to the nearest 500 g), respectively. World Health Organization (WHO) criteria was used to interpret the data, and the Anthro Plus^®^ programme (WHO, Geneva, Switzerland) was used to calculate *z*-scores for individuals under 19 years. 

### 2.4. Dietary Assessment

Dietary assessments and any dietary changes were performed by two inherited metabolic disorders nutritionists (M.F.A. and J.C.R.) in a standardised way. The data from the 24-h recall dietary assessment was transferred to an Excel (Microsoft, Washington, USA) sheet created with nutritional composition values of regular food from the Portuguese Food Composition Tables. The nutritional composition of special low-protein foods and protein substitutes were also added to this database. All macro and micronutrients intakes were also estimated. 

### 2.5. Blood Phe Levels

Median blood Phe levels were measured by fasting blood spots performed at home, which were analysed by tandem mass spectrometry. Most of the patients performed their own blood spots, but in some cases blood spots were taken by parents. Both parents and patients received training from specialist nurses or laboratory assistants on how to do blood spots. Phe levels were collected for a median of 6 months before the first and second dietary assessment (Table 1), with a median of 6 (range 2–20) (assessment period 1) vs. 11 (range 2–23) blood spots (assessment period 2) (Table 2). 

### 2.6. Reassessment of NP Tolerance

Dietary intake was established at baseline assessment and additional NP was introduced gradually in order to maintain blood Phe < 480 µmol/L or until a normal protein intake was achieved. Generally, if there was no change in blood Phe levels after 1 to 2 blood spot samples (1 to 2 weeks), NP was increased by at least one Phe exchange (20 mg of Phe/100 g or around 0.4 g protein), but this varied between individual patients. NP was supplied by plant or animal protein, according to the individual patient choice. This process was repeated providing the blood Phe level remained <480 µmol/L. Individual patient advice and feedback was given by the nutritionist by telephone on the receipt of each blood Phe result. If blood Phe levels remained within range, patients were advised to continue to increase NP intake. 

### 2.7. Ethical Statement

Ethical approval to perform this study was given by the Ethics Committee of Centro Hospitalar Universitário do Porto, EPE, on the 18 May 2015, for the investigation project TNSPKU (Trends in Nutritional Status of patients with phenylketonuria), with the reference 2015.101 (092-DEFI/087-CES).

All patients/caregivers gave written informed consent to participate in this study. This study was also approved by the Faculty Research Ethics and Integrity Committee of the University of Plymouth (Reference Number: 17/18-890). 

### 2.8. Statistics

Analyses were performed using SPSS (version 22, SPSS Inc., Chicago, IL, USA) and R (version 3, R Foundation for Statistical Computing, Vienna, Austria). Continuous data are presented as medians (interquartile ranges) and categorical data are presented as frequencies of counts with associated percentages. Comparisons between continuous variables were performed using *t*-tests with analysis of protein intake and blood Phe levels across assessments carried out using repeated measures ANOVA techniques. A *p*-value < 0.05 was considered statistically significant throughout.

## 3. Results

### 3.1. Subjects

We collected data on 40 patients with PKU with a mean age of 17 years (range 12–29 years). Twenty-four patients were males (60%) and 16 were females (40%). All participants were diagnosed by newborn screening and continuously treated with a low-Phe diet following diagnosis. Ten patients (25%) had hyperphenylalaninemia (HPA), 23 mild PKU (58%) and 7 classic PKU (17%). The severity of the disorder was defined according to Portuguese definitions [16]: HPA: blood Phe ≥150 and ≤360 µmol/L at newborn screening, but >360 µmol/L post newborn screening; mild PKU: blood Phe >360 µmol/L and ≤1200 µmol/L; classical PKU: blood Phe >1200 µmol/L. Table 1 describes patient characteristics across the different types of PKU. All patients were treated with a low-Phe diet together with a Phe-free L-amino acid supplement (*n* = 36) or a low-Phe glycomacropeptide-based protein substitute (CGMP-AA) partially contributing to total protein substitute intake (*n* = 4). Four patients were prescribed additional docosahexaenoic acid (DHA) supplementation, as this was not added to their protein substitute. No other vitamin and mineral supplements were given.

### 3.2. Change in Natural Protein Intake 

The median NP intake significantly increased from the first to the second assessment (35 (23–60) vs. 40 (25–75) g/day, *p* = 0.01, Table 2). Twenty-six of forty patients (65%) increased NP intake by a median of 12 g/day (range 2–42) between both assessments. The remaining 14 patients (35%) did not tolerate any additional NP intake after protein challenge, as it increased blood Phe levels. Four patients increased NP from <20 g/day to ≥20 g/day. Twenty-two of forty patients (55%) tolerated at least 20 g/day of NP on their first assessment. When expressed as g/kg/day, NP intake still increased between first and second assessment (0.77 vs. 0.94 g NP/kg/day, *p* < 0.001). 

**Figure 1:**
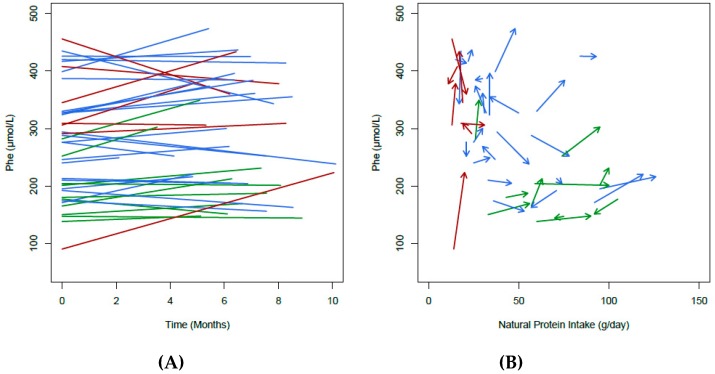
(**A**) Change in blood Phe (µmol/L) over time with fitted regression line and (**B**) pairwise change in blood Phe (µmol/L) and total natural protein (g/day) intake between assessments. Red lines denote subjects with classical PKU, blue lines denote subjects with mild PKU and green lines denote subjects with HPA.

In total, 15% (*n* = 6/40) of patients increased NP intake by at least 50%. The remaining patients (20/40) who were able to increase their NP intake did so by a minimum of 20%. Figure 1 shows both the change in Phe over time, total NP intake and pairwise change in Phe levels. Most (*n* = 19) of the 26 patients who had the highest increase in NP intake were adolescents.

**Figure 2:**
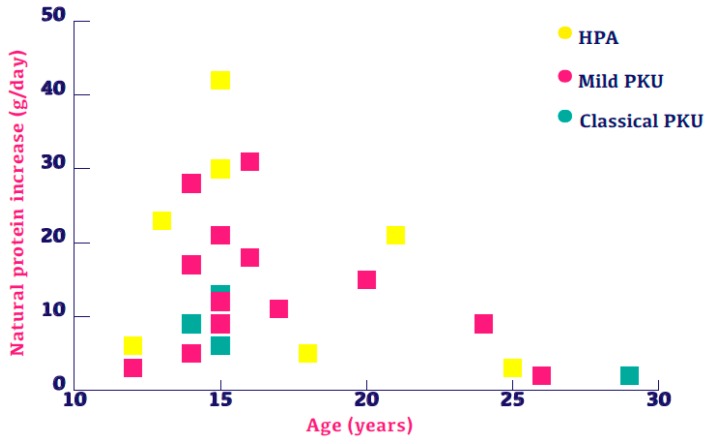
Twenty-six subjects with increased natural protein presented by age and severity of disorder.

**Figure 3:**
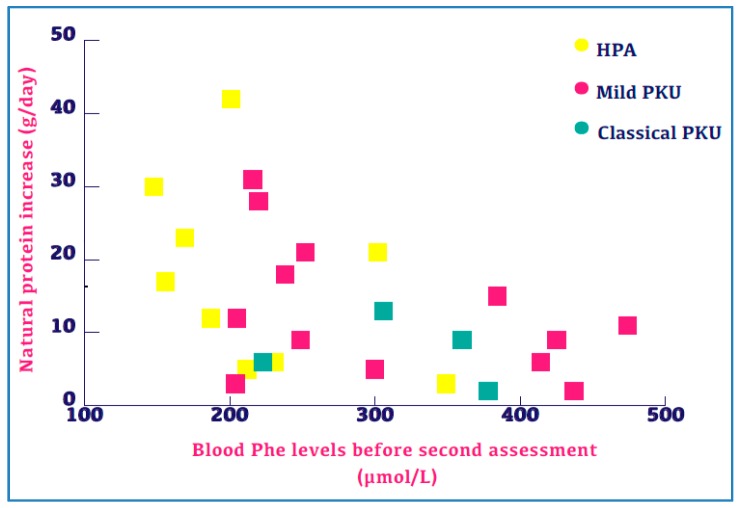
Twenty-six subjects with increased natural protein presented by median blood Phe levels and severity of disorder.

Patients with all forms of PKU severity were able to increase NP (Figure 2) regardless of age. At least half of the patients in each disorder severity were able to increase NP intake by a minimum of 20%. Table 2 outlines the number of patients, their disorder severity and the NP increase. Figure 3 describes NP increase compared with blood Phe levels and disorder severity. Patients with mild PKU have consistently lower Phe levels relative to other patient populations whilst patients with classical PKU have consistently lower NP relative to other patient populations. Linear regression was performed to evaluate the change in protein between assessments, and neither the baseline protein intake (*p* = 0.177) nor the severity of disorder (*p* = 0.611) was associated with the NP increase. 

### 3.3. Source of Natural Protein 

In the patients that increased their NP intake (*n* = 26), 77% used animal sources such as meat, fish, eggs or milk (Table 3). All HPA and 79% (11/14) of the mild PKU patients increased NP from animal protein. In contrast, most patients with classical PKU increased NP from plant sources (3/4) using foods such as bread, pasta and potatoes. 

When protein was sourced from animal origin (*n* = 20), the median total protein prescription (NP plus protein substitute) was 68 g/day (range, 15–126 g) on the second assessment. In comparison, the group with only vegetable protein sources (*n* = 6) had a median total protein prescription of 23 g/day (range, 20–48) on the second appointment. 

### 3.4. Protein Substitute 

The median protein equivalent from protein substitute intake decreased between the first and second assessment (0.82 vs. 0.75 g/kg, *p* = 0.01). Most patients (*n* = 36) took a Phe-free L-amino acid supplement only, but *n* = 4 (of 40) took a combination of CGMP-AA and L-amino acid supplements.

### 3.5. Energy Intake

The median energy intake (kcal/day) (*n* = 40) did not change between the two assessments (2371 vs. 2365, *p* = 0.759). In the patients with increased NP tolerance, their median energy intake also remained unchanged (2335 vs. 2400, *p* = 0.054). 

### 3.6. Blood Phe Control

Median blood Phe control remained stable and within the target range of the Portuguese guidelines [6] when comparing the results from the first and second assessment (279 vs. 288 μmol/L, *p* = 0.06) (Table 2). Statistical modelling of the change in Phe was performed using analysis of covariance (ANCOVA) techniques and showed that both baseline blood Phe and change in Phe over time differ by disease categories (Figure 1B). Specifically, both HPA and mild PKU demonstrated a per month decrease in blood Phe levels (−11.6 (9.2) μmol/L and −10.9 (6.1) μmol/L, respectively), whereas the classical PKU patients observed a per month increase of 9.0 (10.22) μmol/L, although these differences did not meet statistical significance (*p* = 0.153).

### 3.7. Changes in Anthropometric Measurements

During a median of 6 months (study period), weight increased from (*n* = 40) a median of 52.5 vs. 58.0 kg, *p* < 0.001 and height from a median of 161.0 vs. 163.1 cm, *p* < 0.001). 

In patients <19 years (*n* = 29), height increased from 159.2 to 163.0 cm, *p* < 0.001; *n* = 19/29 increased NP intake. No differences were found in this group regarding median height *z*-sores (−0.33 vs. −0.07, *p* = 0.933), BMI (19.6 vs. 21.3 kg/m^2^, *p* = 0.06) and BMI *z*-scores (0.00 vs. 0.37, *p* = 0.405). The growth of patients is presented in Table 4.

## 4. Discussion

The main finding of this study is that 65% of patients with PKU aged ≥12 years were able to tolerate more NP than prescribed when challenged. The majority of patients were adolescents, and 50% of all the patients were able to increase NP intake by a minimum of 20%. Interestingly, patients with different disorder severities all tolerated more NP, but in the classical PKU group extra NP was sourced mainly from plant sources. Median blood Phe remained unchanged in patients who tolerated additional NP intake. These results are very encouraging, particularly as the current Portuguese upper target blood Phe levels (maximum 480 µmol/L) [3] in patients >12 years are stricter than the European PKU guidelines [6]. 

The challenge with extra dietary protein was performed in preparation for a BH4 loading test in order to examine in a controlled way the maximum Phe tolerance of patients before a trial with BH4 [17]. Even in patients who were proven not to respond to BH4, many were still able to increase NP intake without deterioration of blood Phe control. A median NP increase of 12 g/day natural protein is equivalent to three slices of regular bread or 60 g of cheese daily. Therefore, this provided an important improvement in both the amount and quality types of NP offered, which possibly would ease some of the dietary burden, improve patient ability to socialise more easily and contribute to better dietary adherence.

We found the higher increases of NP intake in adolescent patients. In many clinics, adolescents are less adherent with diet therapy, so it is difficult to assess their definitive dietary Phe tolerance and the impact of growth on increasing Phe tolerance. There is a clear need to assess maximum NP tolerance more regularly as part of routine PKU care. If blood Phe levels are consistently maintained within target blood Phe levels for at least 3 months (i.e., 120 to 480 µmol/L) an increase in Phe intake should be considered. In our study, patients had normal body weight, and their NP tolerance increased irrespective of individual weight. In contrast, MacLeod et al. showed in overweight adults [9] that Phe tolerance was increased only when expressed relative to ideal and not actual body weight. These data highlight the importance of body composition (e.g., lean body mass) in interpreting NP tolerance in PKU [18].

In our study, most of the HPA and mild patients with PKU obtained additional NP from animal protein sources (e.g., dairy products, meat and fish), while classical patients with PKU obtained 75% of their additional NP from plant protein sources (e.g., bread, pasta and potatoes). Plant protein has a low digestibility (50–80%) associated with resistant fibres in plant cell walls, food processing, heat or the presence of anti-nutritional factors, which may increase losses of endogenous protein at the terminal ileum [19]. This may suggest that perhaps it would be beneficial if a percentage of NP allowance was from animal sources. It is also probable that the digestion and absorption kinetics of plant protein is different from that of protein in milk and yoghurt, thereby affecting post-prandial Phe concentrations. Furthermore, most of our patients were given NP as cow’s milk together with protein substitute in order to avoid patients developing a taste preference for other NP sources. It is possible that administering NP together with a protein substitute may improve protein synthesis, but this still requires further research.

This study has several limitations. The patients with PKU participating in this study had all types of disorder severity, which complicated the interpretation of the results. We believe that the results are reproducible in all variations of PKU patients, but NP tolerance will be more limited in classical PKU. A larger cohort of adolescent and adult patients with classical PKU is necessary to give a definitive estimate of NP tolerance and variability of intake in adulthood. Also, we did not collect data on Phe tolerance in early life and therefore cannot describe changes in tolerance throughout life.

This is a retrospective uncontrolled study. The accuracy of assessment of NP intake may have been reduced by dietary methodology procedures, as all assessments were taken in a routine clinic using 24-h recall methods. It is possible that the NP intakes could have been over- or underestimated, and patients may have overestimated their adherence with daily protein substitute prescription in particular. A controlled interventional study would help improve the accuracy of data collection. 

## 5. Conclusions

In conclusion, it seems that many patients with PKU with all types of disorder severity tolerate more NP than prescribed, and in some cases the increase was at least 50%. This extra NP may positively influence a patient’s nutritional status and quality of life, as eating more natural food is likely to positively affect the general health status of the patient. It is clear that most of our patients were over-restricted in NP allowance. Therefore, it is beneficial to assess maximum NP tolerance at periodic intervals in clinical care, as different variables may affect tolerance. There is very little information on how Phe tolerance changes with increasing age, so more data is needed to clearly show how NP tolerance changes from childhood until adulthood.

## Figures and Tables

**Table 1:** General subject characteristics.

Covariate	Level	Classical PKU	HPA	Mild PKU	Total
Total		**7**	**10**	**23**	**40**
Gender	F	2 (12%)	3 (19%)	11 (69%)	16
	M	5 (21%)	7 (29%)	12 (50%)	24
Age (months)	median (IQR)	190 (169.0, 243.5)	185.5 (168.3, 214.5)	190 (179.5, 270.5)	190 (175.5, 256.0)
Diagnostic level (blood Phe in mg/dl)	median (IQR)	25.2 (21.65, 27.75)	4.65 (4.05, 5.18)	11 (7.65, 15.00)	9.95 (5.88, 16.00)
Months between assessments	median (IQR)	6 (5.5, 8.0)	6.5 (5.3, 7.0)	7 (5.5, 7.0)	6.5 (5, 7.3)

Abbreviations: HPA: hyperphenylalaninemia; PKU: phenylketonuria; IQR: interquartile range; F: female; M: male.

**Table 2:** Anthropometric measurements, dietary intake data and blood Phe levels at baseline (visit 1) and visit 2.

Covariate	Level	Visit 1	Visit 2	Total
Weight (kg)	median (IQR)	52.50 (44.86, 68.63)	58 (47.75, 69.88)	54.25 (45.38, 69.00)
Height (cm)	median (IQR)	161.0 (155.5, 167.8)	163.1 (157.6, 169.1)	162 (156.0, 169.0)
Height *Z* score	median (IQR)	−0.33 (−0.84, 0.29)	−0.07 (−0.88, 0.2)	−0.27 (−0.88, 0.27)
BMI (kg/m^2^)	median (IQR)	21.05 (17.98, 23.63)	21.60 (18.28, 24.23)	21.30 (18.15, 24.03)
BMI *Z* score	median (IQR)	0.00 (−0.74, 1.05)	0.37 (−0.91, 1.43)	0.28 (−0.76, 1.20)
Natural Protein (g/day)	median (IQR)	35.0 (23.5, 60.0)	40.0 (25.5, 74.4)	35.0 (24.0, 70.3)
Natural Protein (g/kg/day)	median (IQR)	0.66 (0.44, 1.04)	0.71 (0.40, 1.14)	0.70 (0.42, 1.06)
Phe * (mg/kg/day)	median (IQR)	32.8 (21.9, 51.9)	35.3 (20.0, 57.1)	35.0 (20.9, 53.1)
Protein equivalent intake from low-Phe/Phe-free protein substitute (g/day)	median (IQR)	45.0 (27.0, 55.4)	41.5 (19.5, 54.8)	44.8 (24.0, 55.1)
Protein equivalent intake from low-Phe/Phe-free protein substitute (g/kg/day)	median (IQR)	0.82 (0.51, 1.07)	0.75 (0.34, 0.99)	0.82 (0.48, 1.04)
Energy intake (Kcal/day)	median (IQR)	2371 (2200, 2536)	2365 (2210, 2603)	2365 (2202, 2575)
Blood Phe Levels (μmol/L)	median (IQR)	279 (194, 328)	284 (210, 375)	279 (203, 360)
Number of blood spots	median (IQR)	6 (4, 9)	11 (7, 14)	9 (5, 12)

Abbreviations: BMI: body mass index; PKU: phenylketonuria; IQR: interquartile range; F: female; M: male. * Phe calculation was based on an estimate that 1 g natural protein yields 50 mg of Phe when phenylalanine analysis of individual foods was unavailable.

**Table 3:** Number of subjects with natural protein increase by disorder severity.

**% Nutritional Protein Increase**	**Total**	**HPA**	**Mild PKU**	**Classical PKU**
Minimum 20% natural protein increase	20/40 (50%)	5/10 (50%)	11/23 (48%)	4/7 (57%)
Minimum 50% natural protein increase	6/40 (15%)	3/10 (30%)	1/23 (4%)	2/7 (29%)
**Source of extra natural protein**	**Total**	**HPA**	**Mild PKU**	**Classical PKU**
Animal foods: Dairy products, fish and meat	20/26 (77%)	8/8 (100%)	11/14 (79%)	1/4(25%)
Plant foods: Bread, pasta and potatoes	6/26 (23%)	0	3/14 (21%)	3/4 (75%)

Abbreviations: HPA: hyperphenylalaninemia; PKU: phenylketonuria.

**Table 4:** Growth in subjects with PKU aged <19 years.

Sample	Height (cm) (1st vs. 2nd Assessment, *p*-Value)	Height *z*-Scores (1st vs. 2nd Assessment, *p*-Value)	BMI (kg/m^2^) (1st vs. 2nd Assessment, *p*-Value)	BMI *z*-Scores (1st vs. 2nd Assessment, *p*-Value)
Patients <19 years (*n* = 29)	159.2 vs. 163.0, *p* < 0.001	−0.33 vs. −0.07, *p* = 0.933	19.6 vs. 21.3, *p* = 0.06	0.00 vs. 0.37, *p* = 0.405
Patients <19 years that increased natural protein intake (*n* = 19)	161.0 vs. 163.0, *p* = 0.001	0.02 vs. 0.06, *p* = 0.469	20.2 vs. 21.3, *p* = 0.034	0.14 vs. 0.37, *p* = 0.920

Abbreviations: BMI: body mass index.
